# Charge mobility retrieval approach from apparent charge packet movements based on the negative differential resistance theory

**DOI:** 10.1038/s41598-018-24327-w

**Published:** 2018-04-12

**Authors:** Jia Meng, Yewen Zhang, Stéphane Holé, Feihu Zheng, Zhenlian An

**Affiliations:** 1 Department of Electrical Engineering, Tongji University, Shanghai, 201804 P.R. China; 2 Shanghai Key Laboratory of Special Artificial Microstructure Materials and Technology, School of Physics Science and Engineering, Tongji University, Shanghai, 200092 P.R. China; 3 Laboratoire de Physique et d’Étude des Matériaux (LPEM, UMR 8213), CNRS, Sorbonne Universités, UPMC, PSL Research University, ESPCI-Paris, Paris, 75005 France

## Abstract

Space charge migration characteristics play an important role in the evaluation of polymer insulation performance. However, an accurate description of charge carrier mobility in several typical insulating polymers such as polyethylene, polypropylene is currently not available. Recently, with the observation of a series of negative charge packet movements associated with the negative differential resistance characteristic of charge mobility in LDPE films, the extraction of charge mobility from the apparent charge packet movement has been attempted using appropriate methods. Based on the previous report of the successful derivation of charge mobility from experimental results using numerical methods, the present research improves the derivation accuracy and describes the details of the charge mobility derivation procedure. Back simulation results under several typical polarizing fields using the derived charge mobility are exhibited. The results indicate that both the NDR theory and the simulation models for the polyethylene materials are reasonable. A significant migration velocity difference between the charge carrier and the charge packet is observed. Back simulations of the charge packet under several typical polarizing fields using the obtained E-v curve show good agreement with the experimental results. The charge packet shapes during the migrations were also found to vary with the polarizing field.

## Introduction

Since it was recognized that space charge has a non-negligible effect on the insulation performance of polymer materials^1–4^, many researchers have explored space charge effects, obtaining many insights^5–7^. However, the charge packet phenomenon is still not well-understood^8^, this phenomenon originates from the accumulation of massive space charge due to electrode charge injection, charge ionization or charge radiation, which migrates inside the polymers in a packet-like shape. Currently, several aspects of the charge packets are still unclear^9^, such as the packet formation mechanism and charge dynamics during the migration^10^. However, this phenomenon offers an excellent opportunity for the evaluation of charge mobility^11,12^, which is an important parameter for polymers and plays an essential role in assessing the insulation performance and in the investigations of new high-performance materials but is difficult to evaluate directly. Moreover, several recent studies have indicated that charge mobility in insulating materials such as PE and PP has a non-linear dependence on the local electric field^13–16^, making the estimation of charge mobility in these materials more complicated. Thus, a simultaneous measurement of the electric field is necessary to improve the confidence in the obtained mobility estimates. In the present study, based on electron beam irradiation and proper numerical methods, it was attempted to extract the negative carrier mobility characteristic from the space charge distribution measurements instead of the traditional current measurements in the polarized PE films. Although the approach for the generation of charge carriers in the material is similar to the electron beam irradiation method in the TOF technique^17^, building on the observation of the charge displacement current in the previous methods, our method is based on a more direct observation of the phenomena by using a space charge distribution measurement technique known as the laser-induced pressure pulse (LIPP) method^18^. Our study is divided into two parts. In the first stage, apparent space charge migration values under a series of polarizing fields are measured experimentally. The polarizing field is set at a relatively low value to prevent potential ionization during the polarization. Both sides of the samples are covered by charge blocking layers to prevent potential charge injection from the electrodes. This method was shown to be effective for the segregation of charges from difference sources. Then, in the second stage, charge mobility characteristics are retrieved from the experimental results obtained in the first stage.

**Figure 1:**
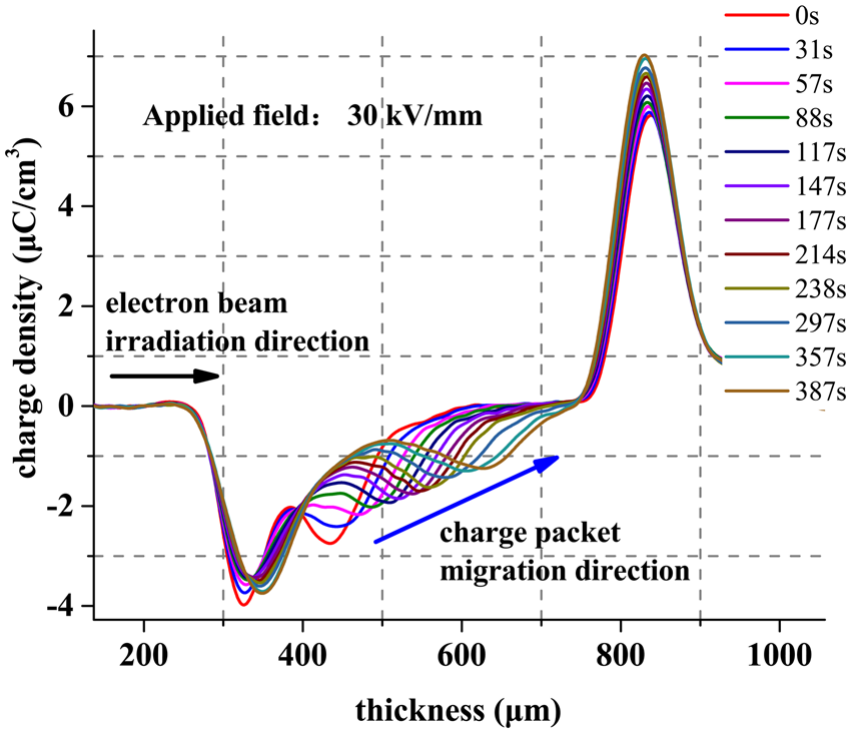
A typical measurement of the charge packet movement in LDPE samples. The applied field is 30 kV/mm^19^.

In the experiment, the samples are hot pressed with LDPE1004 granules. The preparation temperature is 120 °C, and the pressure is 15 MPa. Then, 25-*μ*m-thick PVF(polyvinyl fluoride) films are hot-pressed onto both sides of the samples under the same conditions to prevent charge injections from the electrodes during the subsequent polarizations. Then, both sides of the 500-*μ*m thick samples are coated with 100-nm-thick Al electrodes using thermal evaporation method. Then, the samples are irradiated by an electron beam with the energy of 70 keV and 0.18 *μ*A/cm^2^ for 15 s. After these procedures, the samples are polarized under certain electric fields at 40 °C, and the charge distributions in the samples are measured simultaneously using the LIPP method. A series of charge packet migrations under a broad range of electric fields, from 15 kV/mm to 50 kV/mm, were observed for the first time in our research^19^. This offers an excellent opportunity to elucidate the relationship between charge mobility and the electric field. A typical measurement of charge packet movements in shown in Fig. 1. It can be seen that following electron beam irradiation, a remarkable charge accumulation in a packet shape can be found in the bulk of the sample near the surface of the irradiated side of the sample. Driven by the high field stress, the charge packet gradually drifts to the opposite sample electrode and maintains its shape during the migration. Charge injection from the electrodes is prevented well owing to the charge blocking effect of the PVF films, and negative charges comprise an overwhelming fraction of the internal space charge profiles during the polarizations. The height of the charge packet gradually decreases owing to the trapping effect, and preliminary analyses show that the migration velocity of the packet gradually decreases. After correction of the original measurement signals^20,21^ and simple calculations, the relationship between the charge packet movement velocity and applied electric field was estimated and is reported in^22^ and also shown in Fig. 7 (red) below. A negative differential region for the charge packet speed for electric field *E* ranging from approximately 25 kV/mm to 50 kV/mm was found in the experimental *E* − *v* results. This is in excellent agreement with the NDR model predictions^13^. The key concept of the NDR model is the negative differential charge mobility of the space charge carriers. In the general case, the mobility *μ* of charge carriers is defined as the ratio of the migration speed *v* to the actual electric filed *E* (*μ* = *v*/*E*). However, in the presence of a high amount of charge, such as in a packet, the electric field *E* cannot be considered uniform, and the apparent charge packet velocity differs from that of the charge carriers. Therefore, the velocities of charge carriers and charge packets can be compared only if the packet contains a very small amount of charge. However, an inevitable disadvantage in this case is the difficulty of identifying the position and the quantity of the migrating charge carriers owing to the limited accuracy of the measurement methods. Another possible available approach is to inject a large amount of charges, which is more easily detected, and process the experimental data with an appropriate numerical processing procedure to extract the actual relationship between charge mobility and the electric field. The main difficulty of this approach is that no effective numerical processing has been reported for the extraction of charge mobility from the charge packet movements. To solve this problem, a numerical procedure is proposed and discussed in this paper. The procedure is based on the comparison between the experimental data and charge packet simulations.

## Charge Mobility Retrieval Algorithm

The description of a rough charge mobility retrieval procedure can be found in^22^. The core concept is to generate an *E* − *v* curve describing the relationship between the charge carrier velocity *v* and the local electric field *E*. The curve is then adjusted by comparing the measured signals with the simulated signals through many iterative cycles to make the simulations of the charge packet using the *E* − *v* curve gradually approach the experimental results.

**Figure 2:**
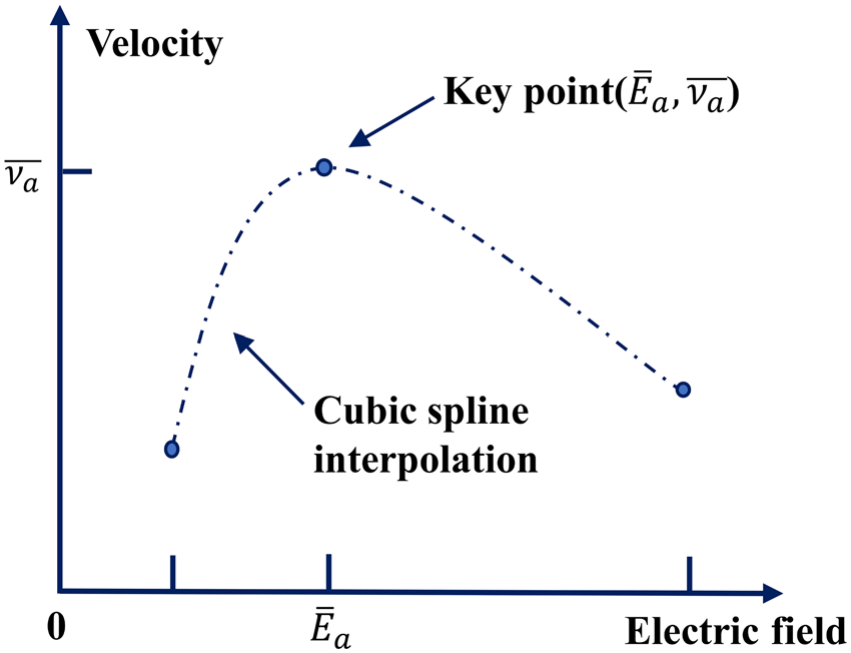
Schematic diagram of the *E* − *v* curve adjusted in the iteration cycle.

**Figure 3:**
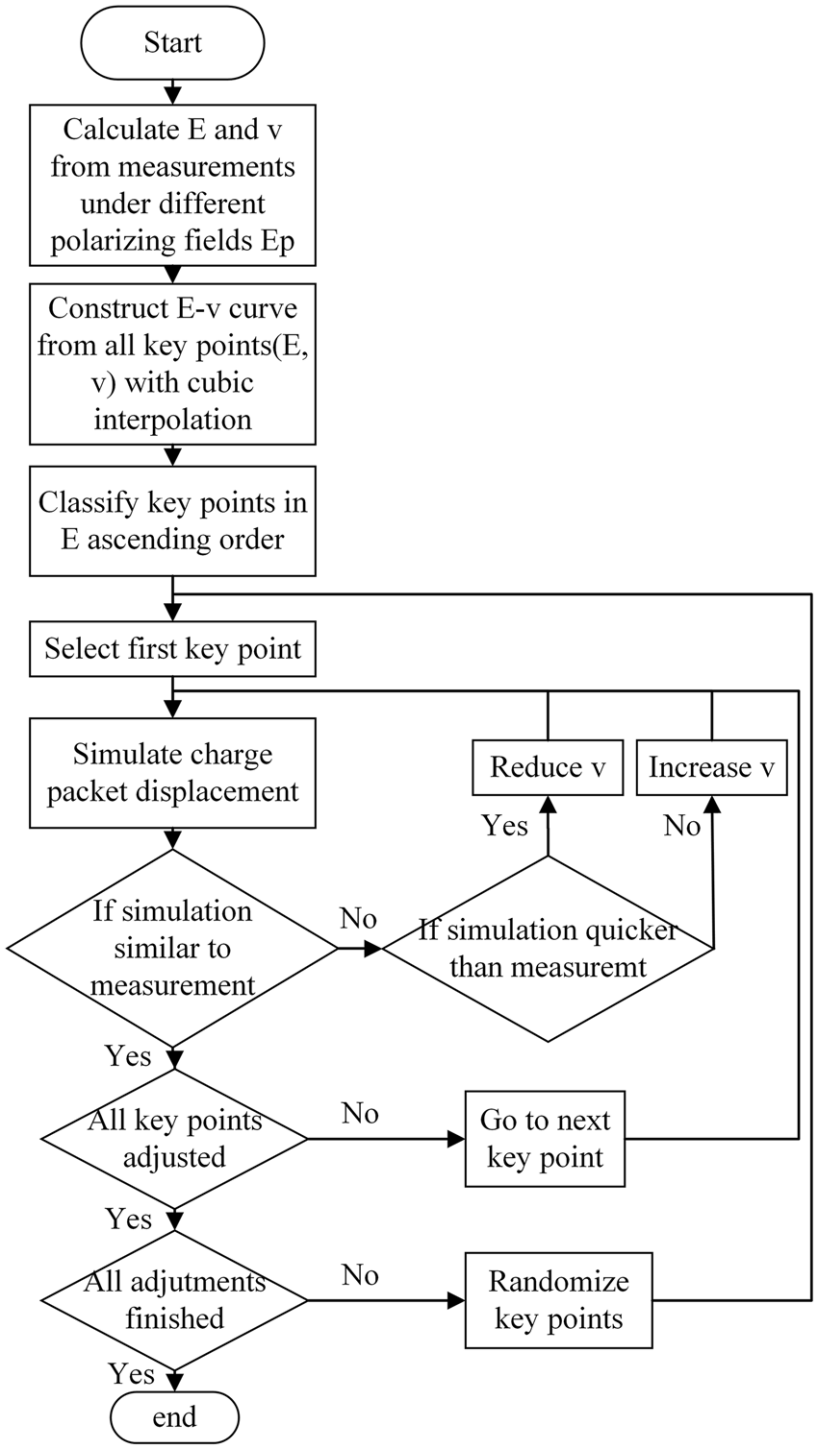
Concise flow chart illustrating the charge mobility retrieval procedure.

First, an initial curve that approximately describes the *E* − *v* characteristics is determined from measurements at given applied electric fields. For this purpose, a large amount of charges is injected inside the samples by electron beam irradiation. When subjected to an electric field, this large amount of charges migrates. Thus, the *E* − *v* curve can be initialized by using the electric field calculated from the measurements and the apparent velocity measured for the large amount of charges. Since the velocity of the charge packet depends on the position, the apparent velocity is calculated from the average velocity obtained throughout the sample. Since the electric field is not uniform throughout the sample, the maximum electric field is used because it corresponds to the local electric field at the edge of the charge packet. The measurement under a given electric field then gives the point on the *E* − *v* curve that we call the key point. With experiments carried out under other electric fields, other key points on the *E* − *v* curve are obtained. Thus, the *E* − *v* curve generated from the experimental results can be treated as an approximation of the real *E* − *v* characteristics. To adjust the *E* − *v* curve, a cubic spline interpolation is used between the key points to ensure that the entire curve is smooth and twice differentiable. If the velocity at one key point is changed, the shape of the curve near that key point is correspondingly changed, while the curve far from the position of the key point is less affected. Therefore, only the charges in the packet subjected to the local electric field near the modified key point are affected by the change of the velocity. To simplify the procedure, the key point is only adjusted with respect to velocity. Therefore, the entire *E* − *v* curve can be adjusted locally by acting on each key point separately to reduce the modulation complexity. A schematic diagram of the *E* − *v* curve adjustments in the iterative cycle is shown in Fig. 2. There are two major stages for the key point adjustments depending on the order in which the key points are treated. The first is performed in increasing order, that is, in ascending order of the electric field. The second is performed using a random order to eliminate possible bias. The adjustment can be decomposed into four steps. In the first step, a key point (*E*_*a*_, *v*_*a*_) and the corresponding polarization electric field *E*_*p*_ in the measurements are determined. In the second step, the charge packet movements are simulated under the determined polarizing field *E*_*p*_ using the current *E* − *v* curve. The migration distances *d* of the simulated charge packet front and the experimental results are compared at the end of the stress under *E*_*p*_, that is, after 30 minutes to 1 hour depending on the apparent charge packet velocity. In the third step, if the difference Δ*d* between the migration distances is lower than the tolerance, the adjustment is considered finished. Otherwise, if the distance of the migration of the simulated charge packet is shorter than the experimental distance, *v*_*a*_ is increased, whereas if this distance is longer, *v*_*a*_ is decreased. In the fourth step, the *E* − *v* curve is recalculated and the second and third steps are repeated until the convergence is reached. To improve accuracy, the adjustment is refined by slightly changing the cost function. Instead of estimating Δ*d* between the beginning and the end of the experiment, the sum of the absolute position differences at the same time between the simulation and the experimental results is used. The entire procedure is repeated for each key point. When all key points have been adjusted, the order of the key points is randomized, and the entire procedure is repeated two or three times. This is done to reduce the influence of the artefacts in the calculation on the results. A concise flow chart illustrating the iterative procedure is shown in Fig. 3.

## Charge Packet Migration Simulations

**Figure 4:**
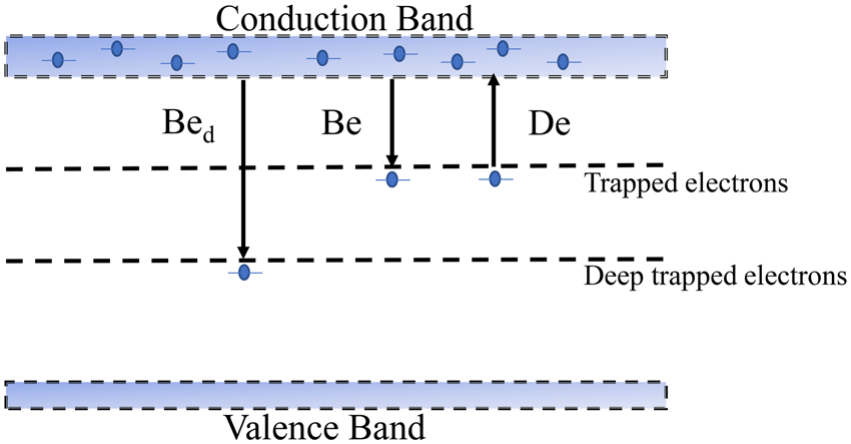
Schematic diagram of the trap level model adopted.

One important factor for the successful retrieval of the charge mobility is the charge packet simulations. Since the end of last century, although many models have been proposed to interpret the charge migration phenomenon, many of them lack universality and can be applied only under certain conditions. Owing to several favourable reports of charge packet simulations, the unipolar charge transport model originating from^23,24^ is adopted to regenerate the charge packet in this work. Meanwhile, the original model is modified to satisfy our experimental conditions: First, since we have introduced PVF films on both sides of the LDPE sample to block the injection effects from the electrodes, the charge-injection part of the model can be neglected. Moreover, because electrons are introduced in the sample by irradiation, and no significant positive charge accumulations are observed during the fast irradiation and the whole polarization, which is similar to G. M. Sessler’s report^25^. Thus only one kind of carrier can be considered if we reasonably neglect the influence of the potential positive charge. As a result, the model can be greatly simplified, and in addition to the positive charge transport process, the recombination process between the different kinds of charges can be neglected. These simplifications effectively help us focus on the negative charge mobility. It should be pointed out that these analyses are for certain situations: charges originate from fast irradiation and charge injections from the electrodes are avoided. Second, based on the analyses of the experimental data, a two-energy-level model is adopted for the charge packet movement simulations. Zhou Tianchun *et al*.^26^ introduced a similar strategy and obtained the desired effects. In addition to the usual shallow trap levels, a deeper trap level is also present. Because the displacements of the charge packet in the experiments are relatively fast (less than 1 hour required for the charge packet to move from one electrode to the other), the de-trapping process for the deeper traps can be neglected. The schematic diagram of the trap levels is shown in Fig. 4. As a result, the three main equations of the charge packet movement model can be expressed based on the Poisson’s equation, the transport equation and the current continuity equation, respectively:

1 $$\frac{\partial E(x,t)}{\partial x}=\frac{{n}_{{c}_{e}}(x,t)+{n}_{{t}_{e}}(x,t)+{n}_{{t}_{{e}_{d}}}(x,t)}{\varepsilon }$$

2 $${j}_{e}={\mu }_{e}(x,t)\cdot {n}_{{c}_{e}}(x,t)\cdot E(x,t)$$

3 $$\frac{\partial {n}_{{c}_{e}}(x,t)}{\partial t}+\frac{\partial {j}_{e}(x,t)}{\partial x}=s(x,t)$$

where *E* is the local electric field, *x* is position, *t* is time, ${n}_{{c}_{e}}$ and ${n}_{{t}_{e}}$ are the charge densities of mobile and trapped electrons, respectively, *j*_*e*_ is the electron current density, and *μ*_*e*_ is the charge mobility. Note that charge diffusion is neglected in the transport equation. Generally, for a steady state 1D problem with one kind of charge carrier, the transport current can be expressed as:

4 $$j(x,t)=\mu \cdot n(x,t)\cdot E(x,t)+D\cdot \frac{\partial n(x,t)}{\partial x}$$

where *D* is the diffusion coefficient which can be related to the mobility by the Nernst-Einstein equation as:

5 $$D=\frac{\mu \cdot k\cdot T}{e}$$

where *k* is the Boltzmann constant, *T* is the temperature, and *e* is the unit charge. Then, Equation (4) can be expressed as:

6 $$j=\mu \cdot n\cdot E+D\cdot \frac{\partial n}{\partial x}=\mu (n\cdot E+\frac{k\cdot T}{e}\cdot \frac{\partial n}{\partial x})$$

For our space charge measurements, $\frac{kT}{e}\cdot \frac{\partial n}{\partial x}$ is of the order of 2.7*10^4^ J/m^4^ whereas *n* ⋅ *E* is larger than 1*10^8^ J/m^4^. Thus, neglect of the diffusion current is a reasonable approximation for the front of the conduction current^27^.

In Equation (3), *s* represents the source term, which represents changes in the local charge density owing to processes other than transport such as the trapping and de-trapping of charges, recombination of hetero charges, etc. It can be expressed as

7 $$s(x,t)={s}_{1}(x,t)+{s}_{2}(x,t)+{s}_{5}(x,t)+{s}_{6}(x,t)$$

Where *s*_1_ and *s*_2_ are the change rates of mobile electron density and trapped electron density, respectively. Irrespective of the combination of hetero charges, the two source terms can be expressed as:

8 $${s}_{1}=\frac{\partial {n}_{{c}_{e}\to {t}_{e}}}{\partial t}=-\,{B}_{e}\cdot {n}_{{c}_{e}}\cdot (1-\frac{{n}_{{t}_{e}}}{{N}_{e}})+{D}_{e}\cdot {n}_{{t}_{e}}$$

9 $${s}_{2}=\frac{\partial {n}_{{t}_{e}}}{\partial t}={B}_{e}\cdot {n}_{{c}_{e}}\cdot (1-\frac{{n}_{{t}_{e}}}{{N}_{e}})-{D}_{e}\cdot {n}_{{t}_{e}}$$

10 $${D}_{e}=\gamma \cdot {e}^{-\frac{{U}_{tre0}-e\cdot a\cdot E}{k\cdot T}}$$

where *B*_*e*_ and *D*_*e*_ are trapping and de-trapping coefficients, respectively, *N*_*e*_ is the electron trap density, *U*_*tre*0_ is the charge barrier height of the shallow traps, *a* is the half width of the potential, *γ* is the escape frequency, *k* is the Boltzmann constant, and *T* is the temperature. It is important to note that (4) does not contain *s*_3_ and *s*_4_ terms because these are used when positive carriers are not neglected. Similarly, irrespective of charge recombination, the two source terms *s*_5_ and *s*_6_ for the deeper trap level are expressed as:

11 $${s}_{5}=\frac{\partial {n}_{{c}_{e}\to {t}_{{e}_{d}}}}{\partial t}=-\,{B}_{{e}_{d}}\cdot {n}_{{c}_{e}}\cdot (1-\frac{{n}_{{t}_{{e}_{d}}}}{{N}_{{e}_{d}}})$$

12 $${s}_{6}=\frac{\partial {n}_{{t}_{{e}_{d}}}}{\partial t}={B}_{{e}_{d}}\cdot {n}_{{c}_{e}}\cdot (1-\frac{{n}_{{t}_{{e}_{d}}}}{{N}_{{e}_{d}}})$$

13 $${B}_{{e}_{d}}=\alpha \cdot {B}_{e}$$

where ${n}_{{t}_{{e}_{d}}}$ is the density of electron captured by the deeper traps, ${N}_{{e}_{d}}$ is the density of the deeper traps, ${B}_{{e}_{d}}$ is the trapping coefficient of the electron in the deeper trap level and *α* is a proportion coefficient. Equations (1) and (2) are solved using the classical finite difference method. For (3), a two-step splitting method is used. In the first step, the source term *s* is discarded, and the rest of Equation (3) is discretized as

14 $${n}_{c}({x}_{i},t+{\rm{\Delta }}t)={n}_{c}({x}_{i},t)-\frac{{\rm{\Delta }}t}{{\rm{\Delta }}x}\cdot [j({x}_{i+1/2},t)-j({x}_{i-1/2},t)]$$

where *x*_*i*_ is the position considered, Δ*t* is the time step, and Δ*x* is the element size. The index *I* ± 1/2 represents a position in the middle of the element beginning at position *x*_*i*_. For the current density *j*, S. Le. Roy^28^ described several algorithms in detail for the constant charge drift velocity. Some modifications to these algorithms are necessary to avoid the problems of flux conservation due to the varying charge carrier drift velocity. In this context, the first-order Upwind method can provide a good balance between the accuracy and the discrete complexity. The detailed discretization schemes can be expressed as

15 $${n}_{c}({x}_{i},t+{\rm{\Delta }}t)={n}_{c}({x}_{i},t)-[{c}_{{x}_{i}}\cdot {n}_{c}({x}_{i},t)-{c}_{{x}_{i-1}}\cdot {n}_{c}({x}_{x-1},t)]$$

16 $${c}_{{x}_{i}}=\frac{{\rm{\Delta }}t}{{\rm{\Delta }}x}\cdot {v}_{{x}_{i}}\cdot {n}_{{x}_{i}}$$

where ${v}_{{x}_{i}}$ is the velocity retrieved from the pre-set *E* − *v* curve. Then, the overall migration process of the charge packet can be simulated with a good predictive performance.

In the second step, the four source terms *s*_1_ *s*_2_ *s*_5_ and *s*_6_ are calculated separately following Equations (8)–(13). Then, the charge density in the different trap levels can be calculated step by step using the following equations:

17 $${n}_{c}(x,t+{\rm{\Delta }}t)={n}_{c}(x,t)+{s}_{1}\cdot {\rm{\Delta }}t$$

18 $${n}_{t}(x,t+{\rm{\Delta }}t)={n}_{t}(x,t)+{s}_{2}\cdot {\rm{\Delta }}t$$

19 $${n}_{c}(x,t+{\rm{\Delta }}t)={n}_{c}(x,t)+{s}_{5}\cdot {\rm{\Delta }}t$$

20 $${n}_{{t}_{d}}(x,t+{\rm{\Delta }}t)={n}_{{t}_{d}}(x,t)+{s}_{6}\cdot {\rm{\Delta }}t$$

The values of the main simulation parameters are given in Table 1. The trapping coefficient^14^, the charge barrier heights^23^ and the half width of the potential^29^ were slightly modified from the values given in previous reports. The choice of the step time Δ*t* and the element size Δ*x* obeys the Courant-Friedrichs-Lewy (CFL) conditions, and Δ*x* varies slightly with the sample thickness.

**Table 1:** Main parameters for the charge packet simulation.

Symbol	Value	Units
Carrier migration speed *v*_*e*_	refer to *E* − *v* curve	*m* ⋅ *s*^−1^
Trapping coefficient *B*_*e*_	0.008	*s* ^−1^
Detrapping coefficient ${D}_{{e}_{d}}$	0	*s* ^−1^
Detrapping barrier height *U*_*tre*0_	0.99	*eV*
Half width of the potential *a*	2.5*10^−9^	*m*
Deep trap densities ${N}_{{t}_{e}}$	100	*C* ⋅ *cm*^3^
Deeper trap densities ${N}_{{t}_{{e}_{d}}}$	100	*C* ⋅ *cm*^3^
Polarizing electric field *E*	15-50	*kV*/*mm*
Temperature	313	*K*
Δ*t*	0.1	*s*
Sample thickness	refer to experimental data	*m*
Δ*x*	vary with sample thicknesses	*m*
Proportionality coefficient *α*	1/15	

**Figure 5:**
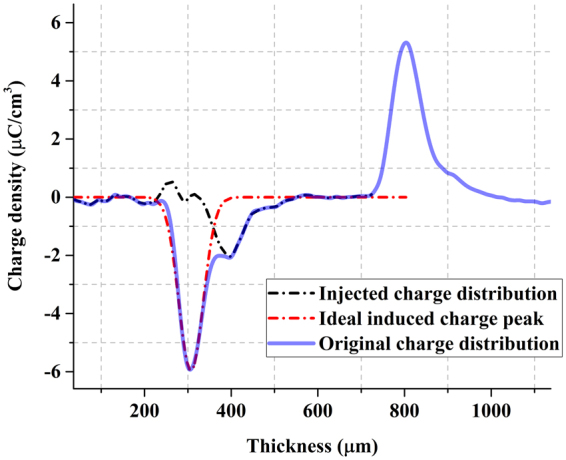
A typical estimation of the injected charge (black short dot line) from a measured signal (blue solid line) using a Gaussian approximation of the induced charge peak (red dash dot line).

To ensure that the simulated charge packet movement is mostly in accordance with the experimental data at each time node, it is necessary to define the initial net charge distribution. In the first measurement at a given electric field, the charge packet is usually not sufficiently far from the electrode to be distinguished from the induced charge peak owing to the measurement spatial resolution. Therefore, it is worthwhile to separate the real injected charge from the entire observed charge distribution. For this purpose, the induced charge peak is approximated by a Gaussian peak. Then, the subtraction of that approximation from the measured signal results in a signal that consists almost entirely of the charge packet. This is illustrated in Fig. 5, which shows that the injected charges are very well detected after the procedure. This result is directly used to initialize the charge distribution in the simulations. This calculation is performed for all applied electric fields.

In the first adjustment pass, the carrier velocity v is adjusted by comparing the positions of the charge packet in the simulation and in the measurement at the end of the polarization. In the second adjustment pass, a finer cost function is used. It compares the position of the charge packet in the simulation and in the measurement at all time nodes. If Δ*d*_*i*_ is the difference between the charge packet position in the simulation and in the measurement at time node *i*, then the cost function *J* is defined at each iteration by

21 $$J=\sum _{i}|{\rm{\Delta }}{d}_{i}|$$

**Figure 6:**
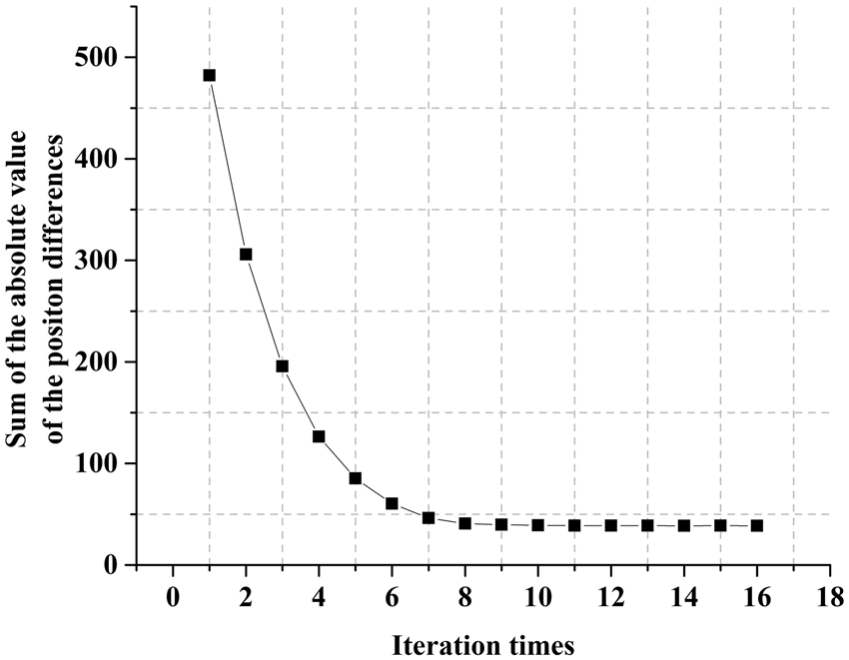
Convergence of the criteria through iterations of a single key node adjustment. The criteria correspond to the sum of the absolute position differences of the charge packet between each simulation and measurement values. After a few iterations, the criterion reaches its minimum.

The considered key point in the *E* − *v* curve is continually adjusted to minimize *J* during the iterations. Then, the difference between the simulated and measured charge packet migration decreases monotonically, as shown in Fig. 6. Once the convergence tolerance is reached, the iterative procedure is stopped, and the next key point is considered. When all key points have been adjusted, the *E* − *v* curve is considered completely retrieved.

## Results and Discussion

**Figure 7:**
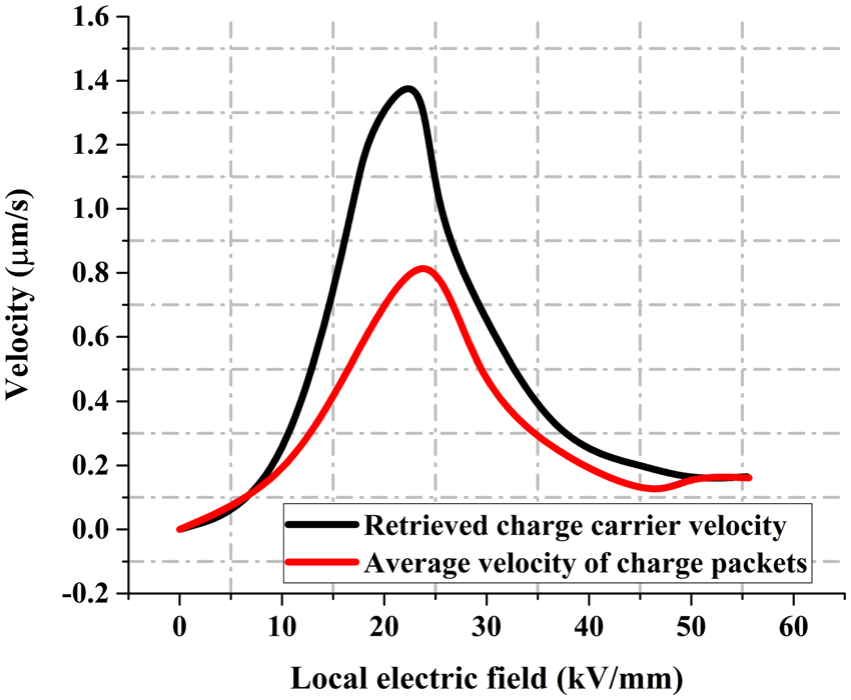
Comparison between original E–v curve and the retrieved curve^22^. Original curve shown in red and retrieved curve shown in black.

**Figure 8:**
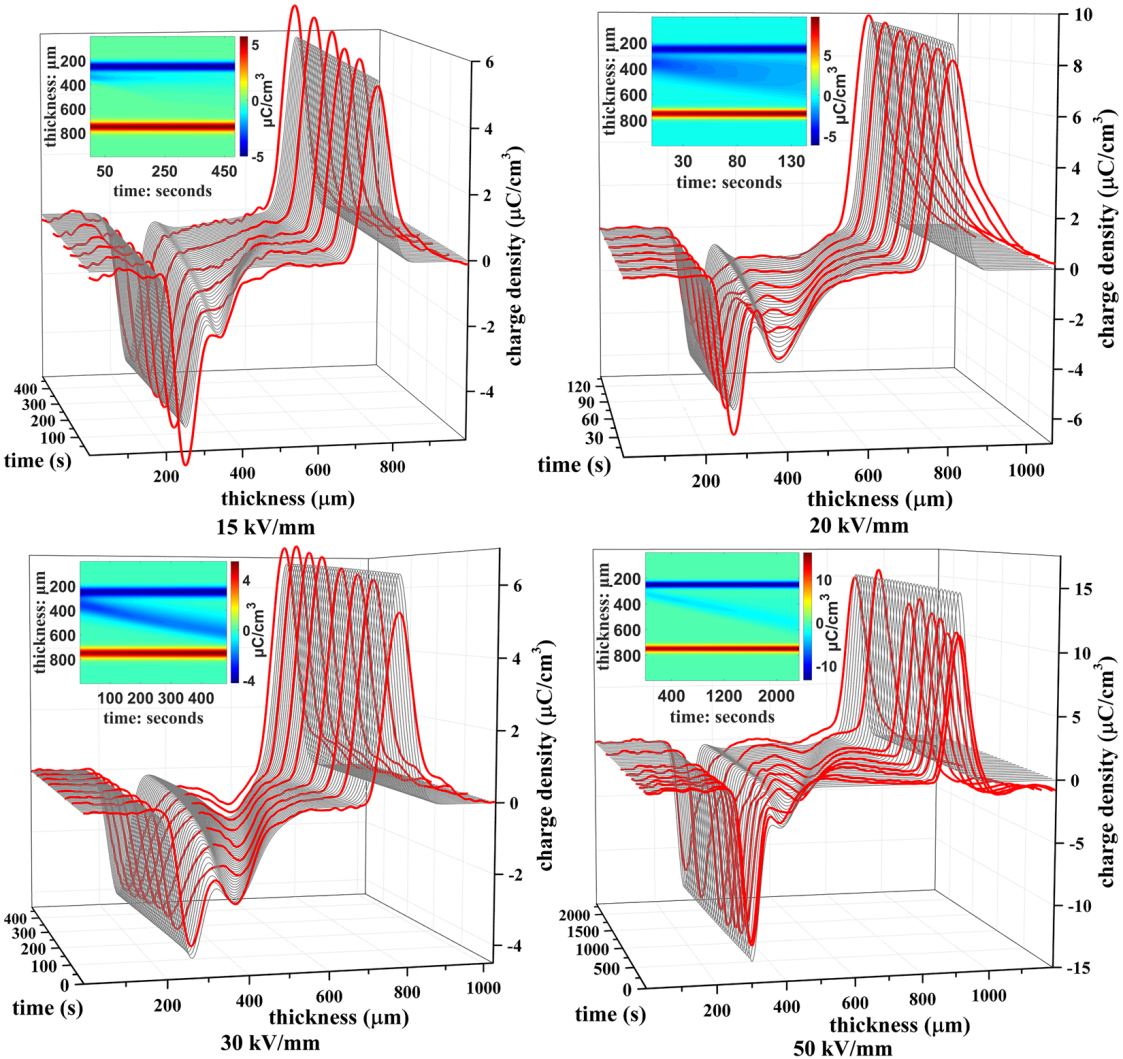
Comparison between experimental and simulated results from the obtained *E* − *v* curve under several typical electric fields. Measurement result are in red and simulation results are in grey. Continuous diagrams of the simulations are shown in the insets.

The procedure described in the previous sections was applied to obtain the *E* − *v* curve of the negative charge carriers in LDPE samples^22^. The comparison between the original E–V curve and the retrieved curve is shown in Fig. 7. Significant differences can be found between the two curves almost across the entire field range. The negative charge mobility ranges from approximately 4 × 10^−15^ m^2^ V^−1^ s^−1^ to 6.5 × 10^−14^ m^2^ V^−1^ s^−1^. For a high electric field, these results are close to the results reported by G. Teyssedre^15^. With the knowledge of the actual *E* − *v* curve, it is now possible to fully reconstruct the movement of the charge packet inside the sample. For this purpose, the same simulation parameters are used as in the retrieval algorithm. Four typical comparisons of simulated and experimental results are shown in Fig. 8. The red lines represent the measured charge distribution at the various times. The charge packet migration is clearly seen in these measurements. The superimposed gray lines represent the simulated charge distributions at a series of time nodes using the retrieved *E* − *v* curve. It can be seen that both experimental and regenerated results show quite similar trends. Both the position and shape of the charge packet are almost identical at all times. This provides strong evidence for the consistency of the retrieved charge mobility and of the validity of the procedure used to obtain the charge mobility from the measurements.

The continuous diagrams of charge density under the corresponding field are shown in the inset of each subgraph. In all continuous diagrams, the charge packet can be clearly identified at the beginning (left part) of the electric field application. After a while, the charge packet spreads at low fields, but shows a more clear drift at high fields. This is the consequence of the *E* − *v* curve. Since at low fields the velocity increases with the field, the packet front moves faster than its tail. In contrast, for higher fields, the charge carrier velocity decreases with the electric field, so that the packet is less deformed during its drift. In addition, more trapping decreases the mobile charge density at low fields. This increases the difficulty in recognizing the charge packet shape under relatively low applied electric fields (15 kV/mm and 20 kV/mm) at the last stage of migration. To summarize, another finding of the present work is that the charge packet shape varies with the applied electric field.

## Conclusion

Based on experimental observations of charge packet migration under various applied electric fields from 15 kV/mm to 50 kV/mm, an accurate *E* − *v* relationship between the velocity of a negative charge and the local electric field in LDPE materials was estimated using numerical methods. The results conform to the NDR assumptions and indicate a significant negative differential mobility zone ranging from 22 kV/mm to 50 kV/mm. A significant difference between the obtained charge carrier velocities and charge packet migration speed is established. Simulations of the charge packet using the retrieved *E* − *v* curve show good agreement with the experimental results. The shape of the charge packet varies with the electric field owing to the non-linear relation between the charge velocity and the electric field.

It should be of great interest to apply and improve the described method further to other experimental charge packet measurements. Further investigations such as studies of the physical nature of the negative differential resistance of the mobile excess electrons will be needed to achieve a clearer picture of the charge mobility in insulating polymers.

### Data availability

The datasets generated during and/or analysed during the current study are available from the corresponding author on reasonable request.
